# Antineutrophil Cytoplasmic Antibodies Associated Vasculitis Presenting As Neuropathy

**DOI:** 10.7759/cureus.57046

**Published:** 2024-03-27

**Authors:** Muskaan Ahlawat, Sachin Shivnitwar, Shubhangi Kanitkar, Akshata Borle, Saipriya Ande, Abhinav Reddy

**Affiliations:** 1 Internal Medicine, Dr. D. Y. Patil Medical College, Hospital and Research Centre, Pimpri, Pune, IND

**Keywords:** remission induction, single organ vasculitis, immunosuppression therapy, acute motor and sensory axonal neuropathy, antineutrophil cytoplasmic antibody (anca) associated vasculitis (aav)

## Abstract

Antineutrophil cytoplasmic antibody-related vasculitis (AAV), is a group of diseases marked by systemic symptoms and severe small vessel inflammation. The three subtypes of AAV are eosinophilic GPA (EGPA), Microscopic Polyangiitis (MPA), and Granulomatosis with Polyangiitis (GPA). The organs that get involved in the disease process are the kidneys and the upper and lower respiratory tracts, with a spectrum of neurological manifestations. Here, we present a case report of a 68-year-old man who came with complaints of tingling and numbness over bilateral lower limbs for two months accompanied by difficulty in walking and bilateral foot drop without any respiratory complaints or involvement of sensory or autonomic system who was diagnosed with AAV (c-ANCA +) on further workup. A sural Nerve biopsy was done for confirmation which was suggestive of chronic, asymmetrical axonal neuropathy with perivascular inflammation, suggestive of vasculitic neuropathy. The patient had no other organ involvement. The patient was started on glucocorticoids and cyclophosphamide therapy for 6 cycles after which his symptoms and quality of life improved drastically.

## Introduction

Blood vessel inflammation and destruction are hallmarks of the clinicopathologic process of vasculitis. Usually, the vessel lumen is damaged, which results in ischemia of the tissues the affected vessel supplies. This process may lead to a wide range of diverse symptoms since it can affect blood vessels of any kind, size, or location [1]. Granulomatosis with polyangiitis (GPA), microscopic polyangiitis (MPA), and eosinophilic GPA (EGPA) are the three subtypes of antineutrophil cytoplasmic antibody-associated vasculitis (AAV). Ninety-five percent of individuals with polyangiitis and granulomatosis have upper respiratory tract involvement [1]. Patients frequently exhibit severe upper respiratory tract symptoms, including purulent or bloody nasal discharge, paranasal sinus discomfort and drainage, and nasal mucosal ulcers [1]. Clinical characteristics of GPA and MPA are similar due to their propensity to affect tiny vessels. The nonspecific symptoms of multisystem disease, such as fever, malaise, anorexia, and weight loss, are frequently seen in patients with eosinophilic granulomatosis with polyangiitis (also known as Churg-Strauss). The second most frequent symptom, mononeuritis multiplex, affects as many as 72% of patients. Up to 61% of patients have allergic rhinitis and sinusitis, which are frequently seen early in the course of the illness. Here, ischemic occlusion of the vasa nervosum-that is, blockage of the tiny blood arteries supplying the nerves-is the cause of the neuropathy [2]. Due to their increased vulnerability to ischemic injury, large myelinated sensory and motor fibers are usually impacted [2]. Neurological manifestations can also be present in the form of mononeuritis multiplex, sensory neuropathy, cranial nerve dysfunction, sensorineural hearing loss, and space-occupying lesions [3]. Immunosuppression remains the mainstay therapy for remission in cases of AAV. The AA usually involves the lung as the major organ. In the following case, patient had no respiratory symptoms and pulmonary involvement was ruled out by doing a CT of the chest.

## Case presentation

A 68-year-old male, cobbler by profession, known case of hypertension and newly diagnosed diabetes mellitus, on regular medications presented to medicine OPD with complaints of tingling and numbness in bilateral upper limb and lower limb accompanied by weakness in all four limbs for 3 months. The tingling and numbness were insidious on onset and gradually progressive in nature. The patient also developed weakness in all four limbs which was accompanied by tightness of the limbs. The weakness began simultaneously in all four limbs. The weakness progressed to such an extent that he was unable to walk up the stairs, sit up from a squatting position, or walk without support. History of cotton wool sensation over the feet was also present. Slippage of chappal with knowledge was experienced by the patient. There was no history of difficulty in walking in the dark room or during nighttime. Over a span of 10 days, the patient’s complaints progressed to such an extent that he was unable to make food bolus or eat by himself. Patient was able to comb his hair, move side to side on and the bed, sit up by himself on the bed. There was no history of bowel, or bladder incontinence, headache, back pain, trauma, altered sensorium, loose stools, vomiting, or involuntary movements. There was no history of cold, cough, weight loss, seasonal allergies, blood in sputum, pain abdomen, vomiting, and or loose stools.

Upon general examination, the patient was conscious, alert, and oriented to time, place, and person. All vital including blood pressure, pulse rate, respiratory rate, oxygen saturation were within normal limits. On neurological examination, bilateral foot drop (right more than left) was present. Higher mental function was normal, cranial nerve examination was unremarkable. On motor system examination, bulk measurements in bilateral upper limb and lower limb showed no muscle wasting or atrophy. the was normal in bilateral upper limbs with hypertonia in bilateral lower limbs. Power was normal in bilateral upper limb (5/5) with poor hand grip bilaterally. Power in the ankles during dorsiflexion was 2/5 bilaterally whereas it was 1/5 bilaterally during plantar flexion. Power at and knee joint and hip joint was normal. Deep tendon reflexes including tricep jerk, bicep jerk, supinator jerk were normal which was Nind’s scale grade 2 bilaterally. Knee jerk was exaggerated (grade 3) bilaterally and ankle reflex was absent bilaterally. Superficial reflexes including abdomen reflex, cremasteric reflexare, and perianal reflex was normal with planters being mute bilaterally. On sensory examination- crude touch, pain, temperature, and fine touch was appreciated by the patient in all four limbs. Joint position sense was impaired in bilateral lower limbs up till the ankle. Vibration sense was reduced in bilateral lower limbs toup to the level of the ankle. He had a high-stepping gait and the Rhomberg sign was positive. Coordination was normal and no cerebellar signs were present. There was no involvement of the autonomic nervous system.

Routine investigations were performed which revealed eosinophilia on complete blood count (absolute eosinophilia count- 1,536). Renal function test was as follows- serum urea- 22, serum creatinine- 0.75, urine protein creatinine ratio-0.46. Rest blood workup was within normal limits. His nerve conduction study was done which was suggestive of asymmetrical sensory motor axonal neuropathy involving all four limbs suggestive of mononeuritis multiplex (Tables 1, 2). On further investigations, Serum antinuclear antibody by immunoflourence (ANA by IF) was positive (1:320). Later his c-ANCA (PR3-ANCA) came out to be positive meanwhile p-ANCA (myeloperoxidase (MPO)-ANCA) was negative.

**Table 1:** Motor Nerve Conduction Study showing asymmetrical loss of motor conduction Table obtained from Nerve conduction study report of the patient from The Department of Neurology, Dr. DY Patil hospital, college and research centre, Pimpri, Pune

Site	Latency (ms)	Duration (ms)	Amplitude	Area
Left Median				
Wrist	3.8	14.8	7.9mV	45.5 mVms
Elbow	8.4	14.7	7.7mV	40.9mVms
Right Median				
Wrist	3.9	6.9	0.7mV	2.5mVms
Elbow	8.8	7.6	0.4mV	2.3mVms
Left Ulnar				
Wrist	Absent	Absent	Absent	Absent
Elbow	Absent	Absent	Absent	Absent
Right Ulnar				
Wrist	3.1	9.6	3.6mV	19.2mVms
Elbow	7.5	18.4	3.5mV	22.2mVms
Left Peroneal				
Ankle	Absent	Absent	Absent	Absent
Popliteal	Absent	Absent	Absent	Absent
Right Peroneal				
Ankle	4.7	7.4	0.3mV	0.4mVms
Head of fibula	11.5	13.1	0.1mV	0.6mVms
Left Tibial				
Ankle	Absent	Absent	Absent	Absent
Head of fibula	Absent	Absent	Absent	Absent
Right Tibial				
Ankle	Absent	Absent	Absent	Absent
Popliteal	Absent	Absent	Absent	Absent

**Table 2:** Sensory Nerve Conduction Study showing asymmetrical loss of sensations Table obtained from nerve conduction study report of the patient as reported by Department of Neurology, Dr. DY Patil Hospital, college and research centre, Pimpri, Pune

Site	Latency (ms)	Amplitude	Area
Left Median			
Wrist	2.8	18.2uV	1.3uVms
Right Median			
wrist	Absent	Absent	Absent
Left Ulnar			
Wrist	Absent	Absent	Absent
Right Ulnar			
Wrist	2.1	11.2uV	1.8uVms
Left Sural			
Sural	Absent	Absent	Absent
Right Sural			
Sural	Absent	Absent	Absent

**Figure 1:**
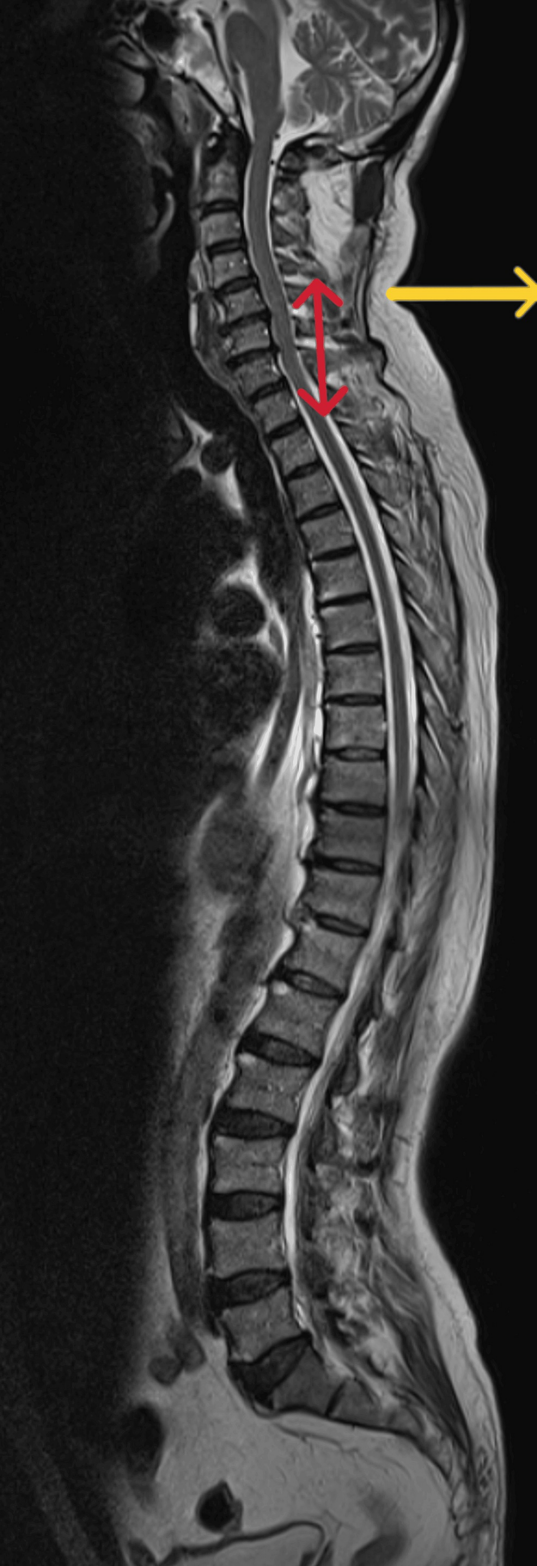
Magnetic resonance imaging of whole spine (axial view) Red arrow indicates posterior disc bulge at C4-C5, C5-C6, C6-C7. Yellow arrow indicates loss of cervical lordosis.

**Figure 2:**
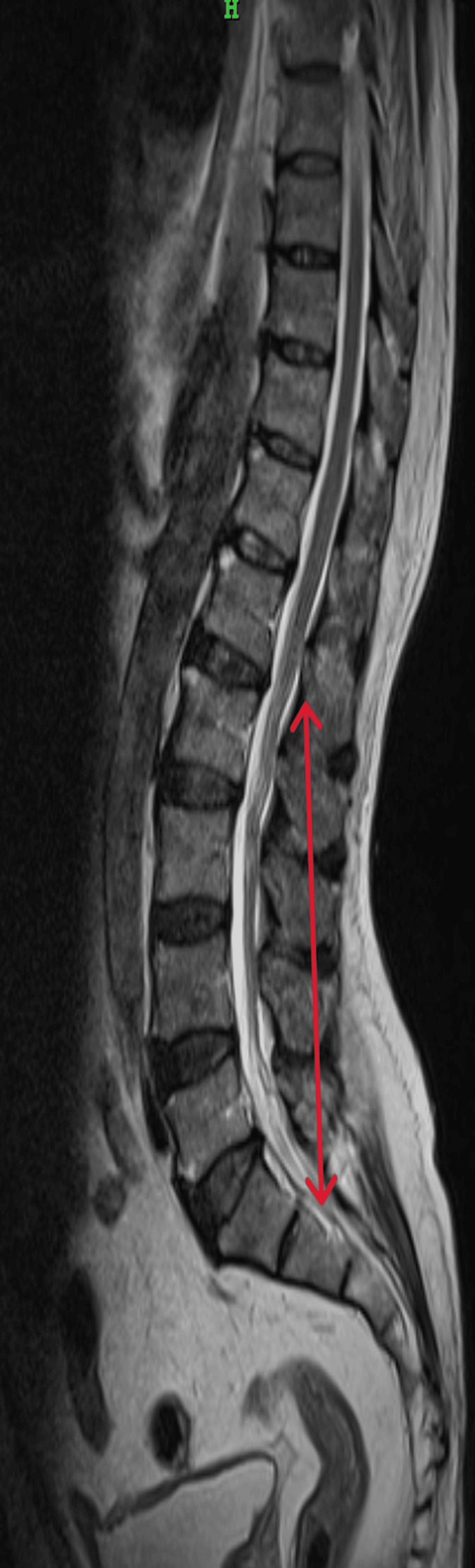
Magnetic resonance imaging of lumbar spine (axial view) Red arrow indicates posterior disc bulges at L2-L3, L3-L4, L4-L5, L5-S1.

MRI whole spine screening was done to rule out any spinal lesions which showed posterior disc bulges noted in C4-C5, C5-C6, and C6-C7 indenting the anterior thecal sac. Posterior disc bulges were also seen in L2-L3, L3-L4, L4-L5, and L5-S1 indenting the anterior thecal sac (Figures 1, 2).

**Figure 3:**
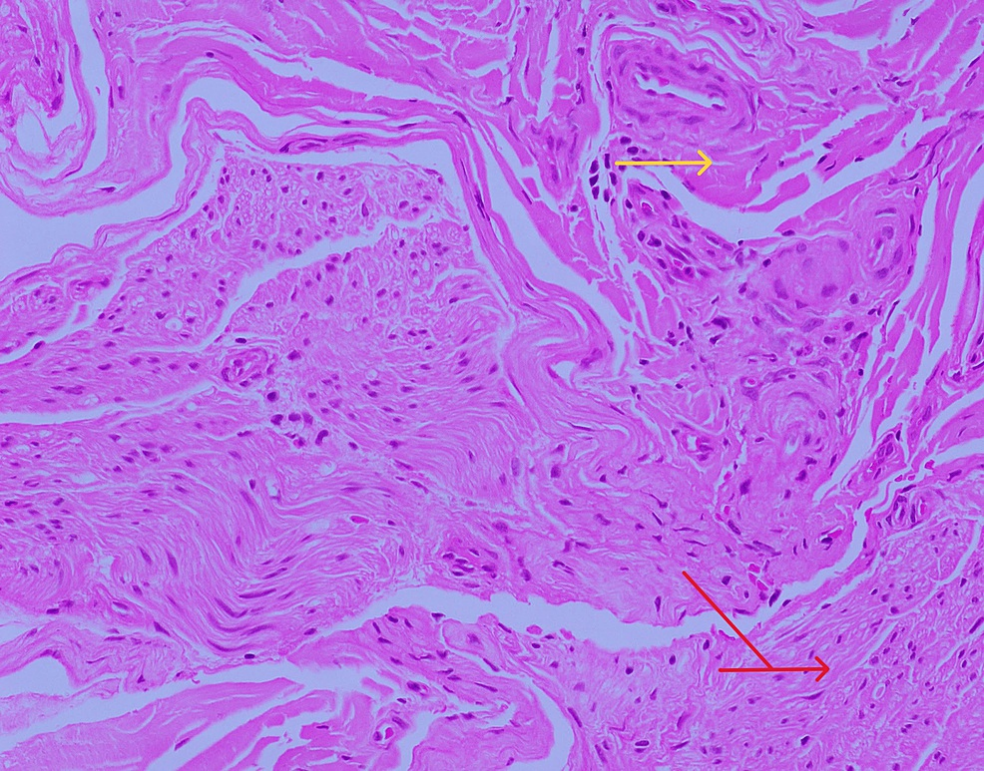
Transverse section of sural nerve, stained with hematoxylin and eosin (H&E) stain (*200x) showing perivascular inflammation Yellow arrow shows perivascular inflammation. Red arrow shows nerve funicles.

**Figure 4:**
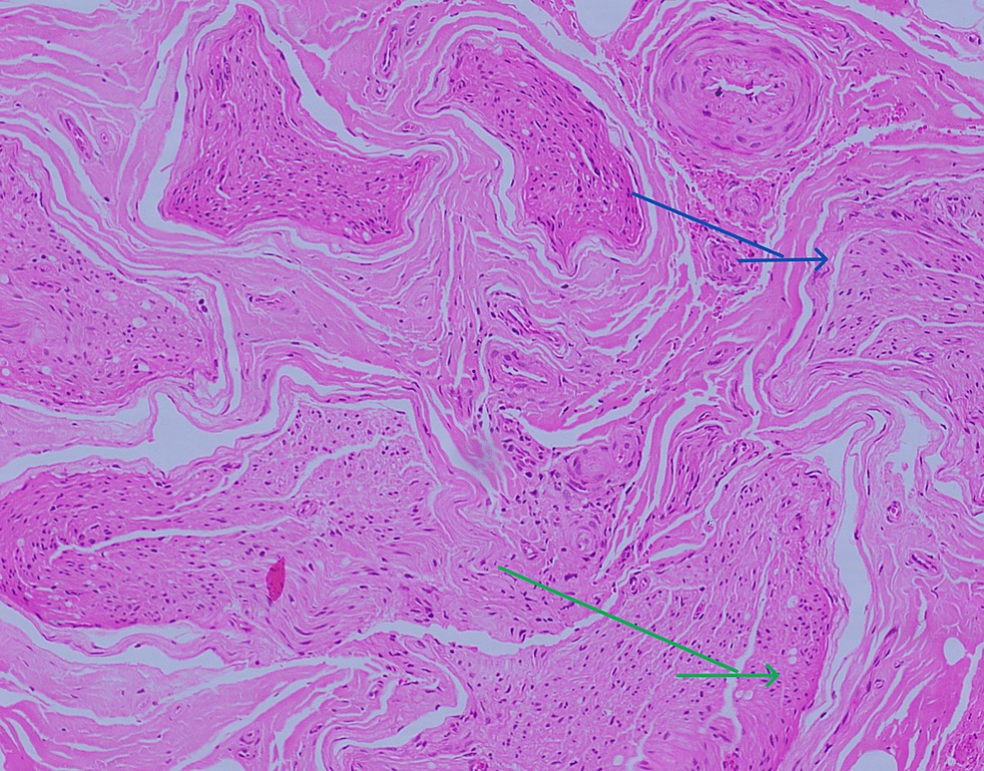
Transverse section of Sural nerve biopsy stained with H&E stain (*100x) showing neovascularization and nerve fiber loss Arrow blue indicates neovascularisation. Arrow green indicates nerve funciles showing significant nerve fiber loss.

**Figure 5:**
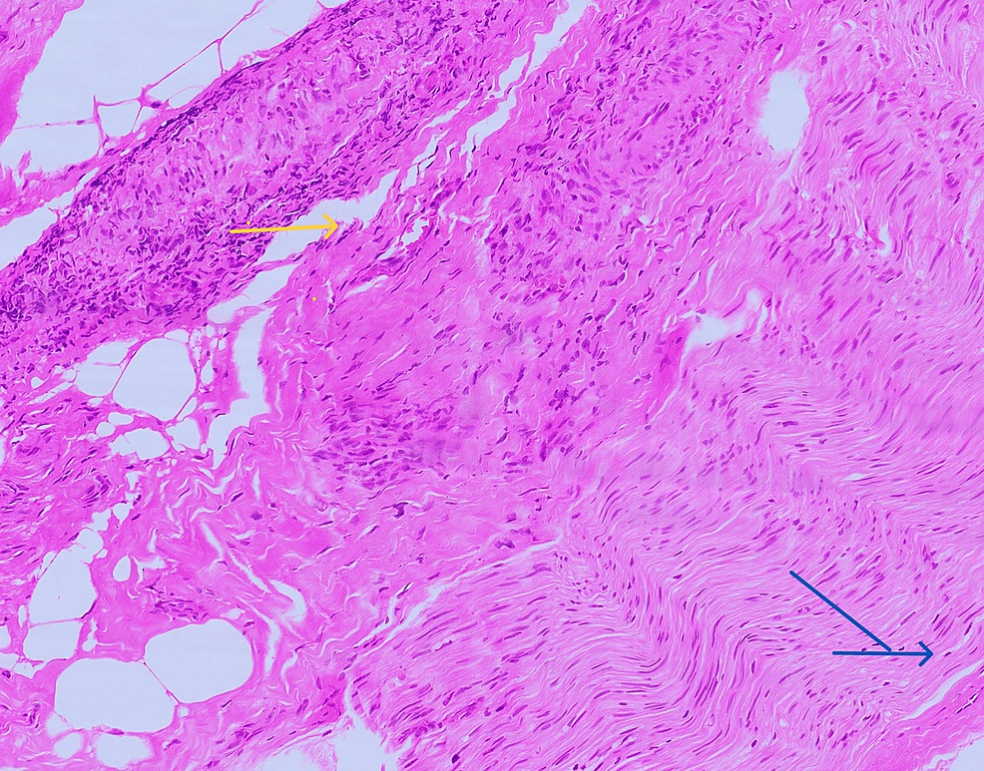
Longitudinal section of the sural nerve stained with H&E stain (*100x) showing blood vessel with dense perivascular inflammation Yellow arrow indicates blood vessel with dense perivascular inflammation. Blue arrow indicates nerve funicles.

**Figure 6:**
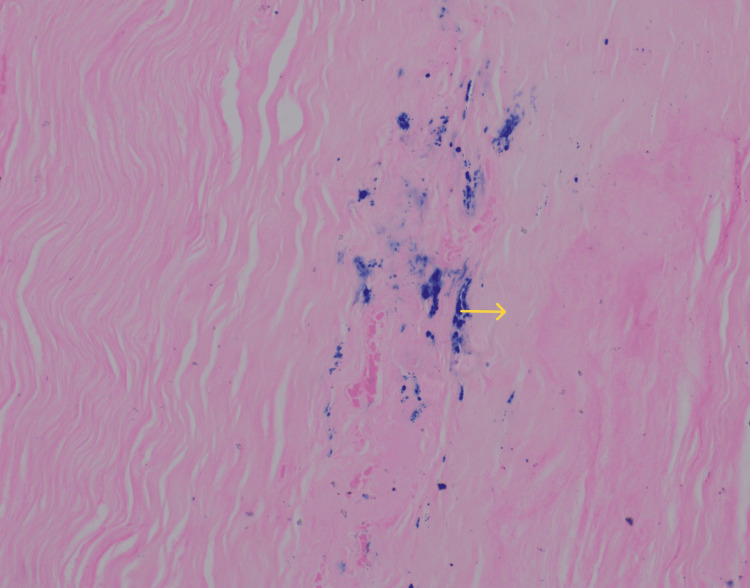
Perl's staining of the nerve fiber (*100x) showing hemosiderin deposits Yellow arrow indicates Hemosiderin deposits.

**Figure 7:**
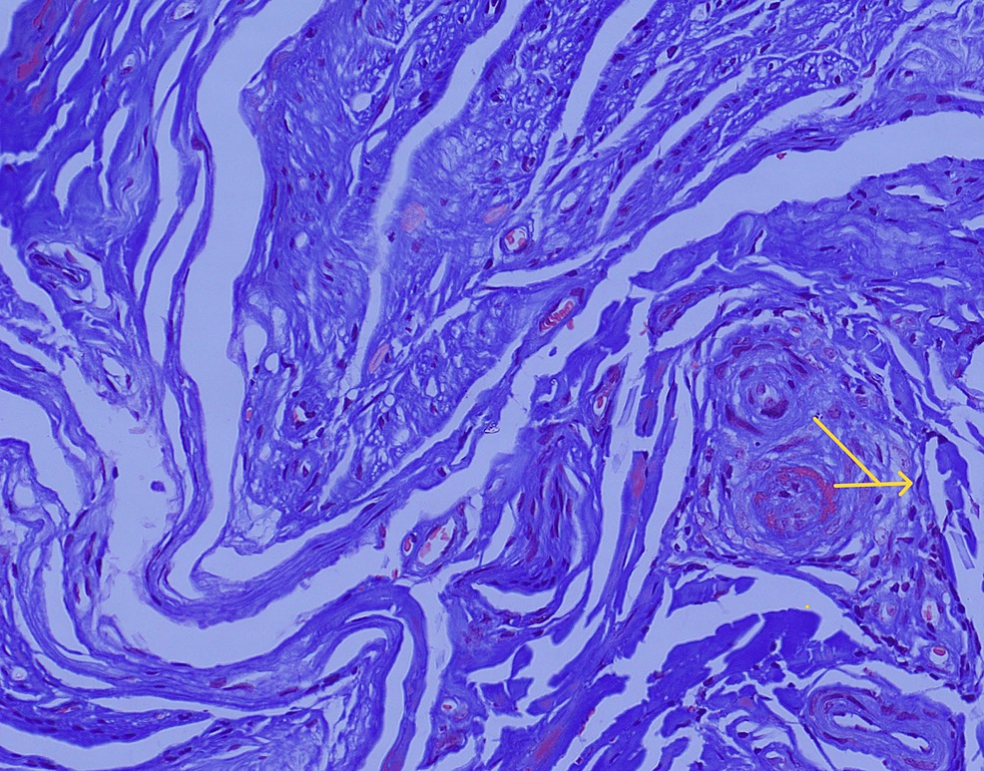
Masson trichrome staining of the sural nerve fiber (*200x) Yellow arrow indicates perivascular inflammation.

MRI brain was done which showed the High-resolutionage-related cortical atrophy. High resolution CT scan of thorax was done to identify any lung lesions, since vasculitis was the first differential diagnosis. Impression of the CT scan was - no obvious parenchyma abnormality. Few calcified lymph nodes noted in pre-paratracheal region. To confirm diagnosis of vasculitis, sural nerve biopsy was performed which was suggestive of chronic, asymmetrical axonal neuropathy with perivascular inflammation, suggestive of vasculitic neuropathy (Figures 3-7).

Taking in account, the clinical presentation, biopsy reports and nerve conduction studies, diagnosis of AAV was made and patient was started on injection Cyclophosphamide and glucocorticoids. Patient has received six cycles of Cyclophosphamide and was initially started on tablet Prednisolone 60mg once daily which has now been tapered off to 10 mg once daily. He is on regular outpatient follow up. Presently, his tingling and numbness has improved, and foot drop has reduced significantly. His quality of life has improved considerably he is carrying out his daily activities without any difficulty.

## Discussion

The 2012 updated Chapel Hill consensus classified AAV as a necrotizing vasculitis that affects tiny vessels and is typically positive for p-ANCA or c-ANCA. Still, it may occasionally also be ANCA negative. It may also have few immune deposits or none at all. EGPA, MPA, and GPA are the three main categories [4]. It mostly affects adults in their later years. Both sexes are equally impacted. Without any indication of specific organ involvement, patients usually present with constitutional symptoms such as fever, loss of weight, muscle pain, and joint pain that can persist for weeks or months. Consequently, GPA is often initially misdiagnosed as inflammatory joint disease, infections, or cancer.

Among the three AAVs, mononeuritis multiplex, sensory neuropathy, cranial nerve dysfunction, sensorineural hearing loss, and space-occupying lesions are the least common in GPA. Up to 15% of individuals have involvement in the peripheral nervous system, with mononeuritis multiplex being the most frequent manifestation. The peroneal, tibial, ulnar, and median nerves are frequently impacted [4].

The ideal duration and dosage of glucocorticoids for ANCA vasculitis are still up for debate. 1-3 g of intravenous (IV) methylprednisolone have historically been used for life- or organ-threatening ANCA vasculitis, and 1 mg/kg of oral prednisone per day has been administered afterward. Prednisone was effectively tapered down by five months in the Rituximab vs Cyclophosphamide for AAV (RAVE) trial. In contrast, other trials continued to administer 5 mg/d of the medication for an additional six months [5]. A combination of low-dose glucocorticoids and azathioprine, rituximab, methotrexate, or mycophenolate mofetil is recommended to maintain remission. Mepolizumab, or anti-interleukin-5 medication, has been effective in treating EGPA patients who are refractory or relapse [6]. Renal, otolaryngological, treatment-related problems (diabetes, osteoporosis, cardiovascular disease, and cancer) and damage escalate with time in AAV patients. At a mean of seven years after diagnosis, almost one-third of patients have at least five items of damage. The most frequently reported treatment-related problems or harm at long-term follow-up were malignancy, diabetes, hypertension, and osteoporosis [7]. Due to the need for ongoing immunotherapies to sustain remission, individuals with AAV are typically impaired. For patients with ANCA-associated vasculitis, trimethoprim-sulfamethoxazole is a crucial therapy adjunct that helps avoid Pneumocystis jirovecii pneumonia. Patients receiving 20 mg or more of prednisone per day for more than a month are advised to have treatment with trimethoprim-sulfamethoxazole, according to a statement from the American Thoracic Society [8].

## Conclusions

AAV usually presents with multisystem involvement predominantly starting from the respiratory system. Through this case report, we intend to bring light to AAV presenting with only nervous system involvement which can be easily missed. Prompt treatment with proper follow-up of the patient can improve the quality of life for the patient and prevent vasculitis-related complications. A lookout for adverse effects of immunosuppression should be noted which may include recurrent infections, sepsis, development of diabetes, hypertension, etc. Even in the absence of respiratory involvement, a diagnosis of vasculitis (AAV) must be considered so that the patient can be started on appropriate treatment.
